# Analysis of Blood Transfusion Request and Utilization Pattern at the Blood Centre of the Tribal District, Dahod, India

**DOI:** 10.7759/cureus.25237

**Published:** 2022-05-23

**Authors:** Kalpesh V Vaghela, Cyrus D Jokhi

**Affiliations:** 1 Pathology, Zydus Medical College and Hospital, Dahod, IND

**Keywords:** wards, audit, red cell concentrate, fresh frozen plasma, platelet rich concentrate

## Abstract

Introduction

Blood is the most commonly donated, vital and essential tissue, saving many lives. It is better to use components instead of whole blood for maximum benefits of blood. Blood is considered a life-saving medicine, so it must be used with precautions as blood and its components can cause side effects such as transfusion-transmitted infections and various transfusion reactions in the recipients. Physicians must therefore be aware of the risk/benefit ratio. Regular internal audits of the blood centres should be conducted to know usage trends. The aim of this study is to analyse the component usage pattern, their demand and the utilization pattern in a tertiary supply centre in Dahod, India.

Material and methods

This is a retrospective, descriptive study conducted over a one-year period from January 1, 2021, to December 31 2021. All data was collected in our blood centre.

Results

5811 blood components were distributed. Red cell concentrate have been utilized the most followed by fresh-frozen plasma and platelet concentrate, respectively. Cryoprecipitate is used the least. Maternity patients benefit greatly from red cell concentrate. The majority of platelet rich concentrate and fresh frozen plasma are utilised by medicine and paediatric departments. The demand for blood is greater as compared to the total collection.

Conclusion

The maternity department receives the majority of the red cell concentrate for the treatment of severe anaemia. Platelet rich concentrate and fresh frozen plasma, on the other hand, are mostly utilised by the medicine department. Different blood components have seasonal variations as well. So, periodic analysis of the usage pattern and need for different blood components at different times also helps maintain blood inventory.

## Introduction

Blood is a specialized body fluid that contains solid and liquid components that play a vital role in delivering oxygen and nutrients, removing waste products and also serving as a primary defence system.

Blood component therapy has gained a lot of interest in recent years due to its advantages over whole blood transfusions, e.g. reducing volume overload in patients, increasing storage life and better patient management. Component therapy was introduced between the 1950s and 1960s to optimize the benefits of all components present within the blood [1]. In India, particularly in tribal areas, there are limited resources of blood and increasing demand, and hence it is necessary to make efficient use of blood [2].

The primary goal of blood centres is to promote high standards of quality in all aspects of production, patient care and service. Regular transfusion audits should be carried out within the blood centre to understand the utilization trends. Unnecessary transfusions may contribute to shortage of blood products and every indication for requisition of blood products should be justified [3]. Appropriate usage of blood components minimizes the burden of transfusion and adverse reactions of transfusions [4]. There are risks associated with blood transfusion just like any other medication. Only use transfusions when the advantages exceed the risks [5].

Otherwise, inappropriate transfusions may result in the waste of precious blood products, other resources such as labour and time, and additional healthcare expenditures. As a result, the needful patients are denied the benefit of blood products, while the injected patients are subjected to transfusion-transmitted illnesses risk and allergic responses as a result of the procedure [6,7].

Thus effective utilization of blood products plays a key role in the quality assurance of transfusion services. It is very difficult for maintaining a healthy balance between continuing blood demand and supply [8]. Interventions should be designed to modify the transfusion practices; thus, periodic revision of existing guidelines is made to enhance the transfusion practices [9]. Thus this study aimed to review the utilization pattern of blood and blood components in a tertiary care hospital.

## Materials and methods

The study was a retrospective descriptive study carried out at the licensed blood centre of Zydus Medical College and Hospital, a tertiary care centre in rural Dahod (Gujarat, India), for a period of one year from January 1, 2021, to December 31, 2021. We collected data of monthly collection and utilisation of blood components from the record books in the blood centre. Details of components issued are documented in the blood centre registers. It included cross-matched and issued blood units. A total of 5811 blood component units were dispensed. We studied utilization (5811 units) of blood and its products in various department of hospital including surgery, gynaecology, orthopaedics and other nonsurgical departments like medicine and paediatrics. The data were entered in an Excel sheet (Microsoft, Redmond, WA, USA) and frequency and distribution of variables were calculated.

## Results

**Figure 1:**
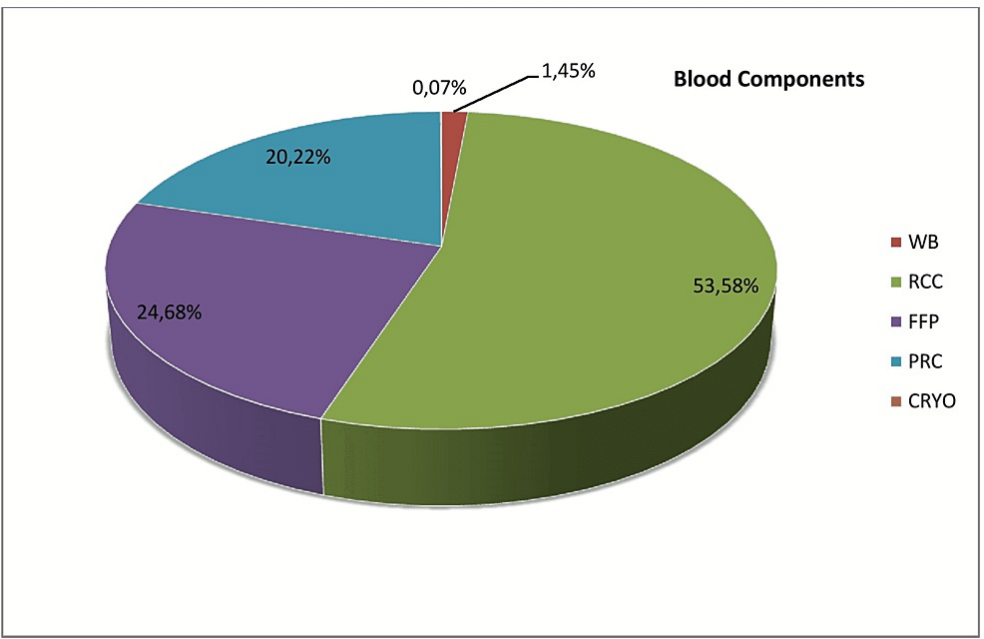
Showing Number of Units of Different Components Issued. WB: whole blood, RCC: red cell concentrate, FFP: fresh frozen plasma, PRC: platelet-rich concentrate, CRYO: cryoprecipitate

**Figure 2:**
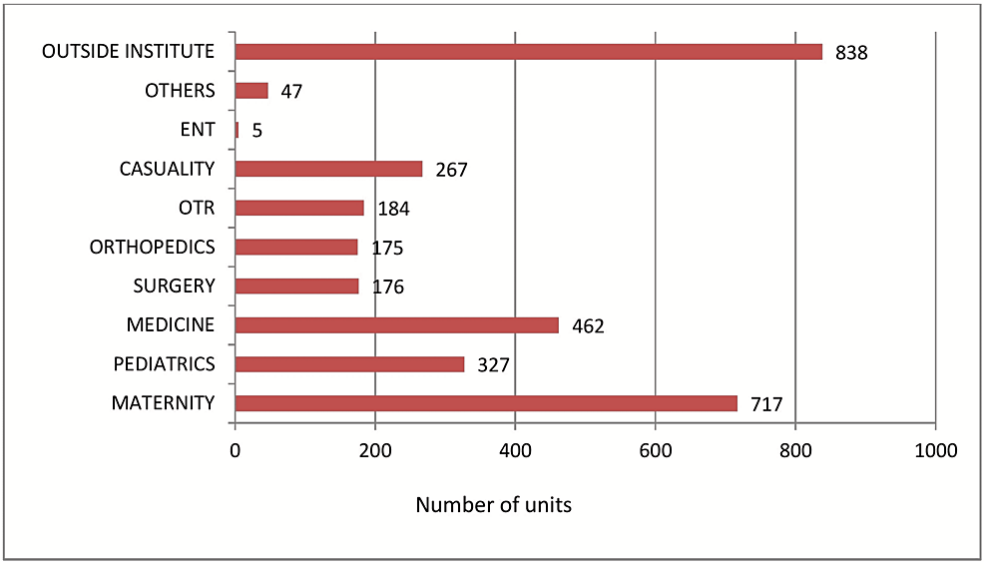
Pattern of RCC and WB Utilised by Different Wards (n=3198). WB: whole blood, RCC: red cell concentrate

**Figure 3:**
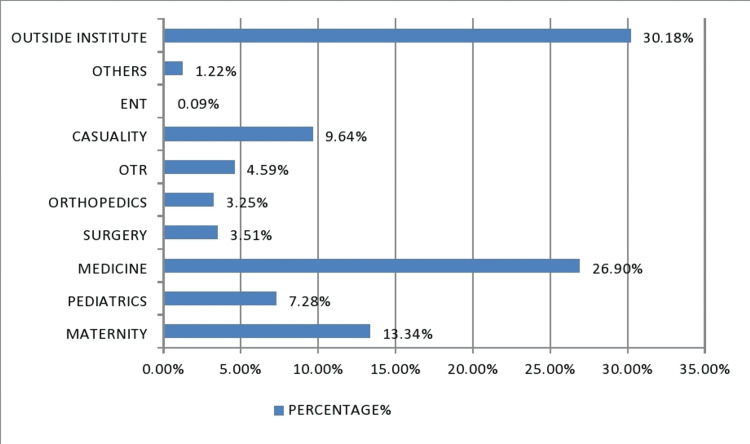
Comparison of various wards for blood components utilization (n=5811).

The total number of units issued in the year 2021 was 5811, out of which red cell concentrate (RCC) was n=3114 followed by fresh frozen plasma (FFP) n=1434, platelet-rich concentrate (PRC) n=1175 and whole blood (WB) n=84 respectively. Cryoprecipitate was the least utilized component with only n=04 units in an entire year (Table 1, Figure 1). The maternity ward majorly benefited from RCC and WB (Table 2, Figure 2). The maximum number of components were issued in the medicine department and the least number of component requirements were seen in the ENT department (Table 3, Figure 3).

**Table 1:** Showing Number of Units of Different Components Issued.

TYPE OF COMPONENT	NUMBER OF UNITS ISSUED	PERCENTAGE %	
Whole blood (WB)	84	1.45%	
Red cell concentrate (RCC)	3114	53.58%	
Fresh frozen plasma (FFP)	1434	24.68%	
Platelet-rich concentrate (PRC)	1175	20.22%	
Cryoprecipitate (CRYO)	4	0.07%	
TOTAL	5811	100%	

**Table 2:** Pattern of RCC and WB Utilised by Different Wards (n=3198). WB: whole blood, RCC: red cell concentrate

Ward	Number of units	Percentage %
MATERNITY	717	22.42%
PEDIATRICS	327	10.23%
MEDICINE	462	14.45%
SURGERY	176	5.50%
ORTHOPEDICS	175	5.47%
OT RECOVERY	184	5.75%
CASUALITY	267	8.35%
ENT	5	0.16%
OTHERS	47	1.47%
OUTSIDE INSTITUTE	838	26.20%
TOTAL	3198	100.00%

**Table 3:** Comparison of various wards for blood components utilization (n=5811).

Ward	Number of units	Percentage %
MATERNITY	775	13.34%
PEDIATRICS	423	7.28%
MEDICINE	1563	26.90%
SURGERY	204	3.51%
ORTHOPEDICS	189	3.25%
OT RECOVERY	267	4.59%
CASUALITY	560	9.64%
ENT	5	0.09%
OTHERS	71	1.22%
OUTSIDE INSTITUTE	1754	30.18%
TOTAL	5811	100%

**Figure 4:**
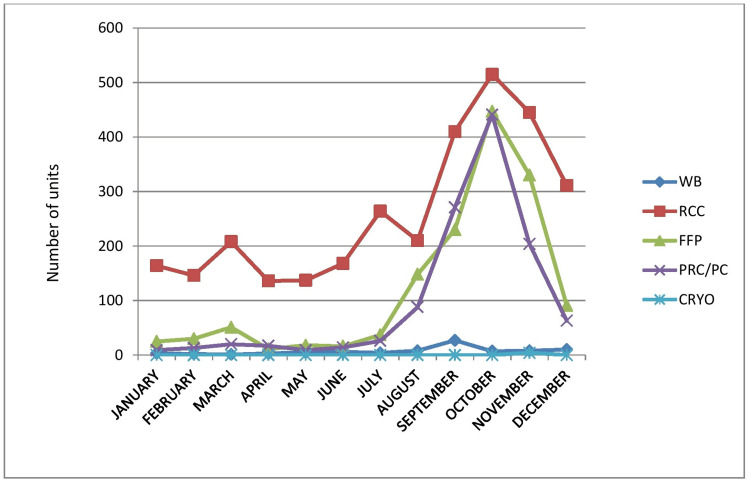
Utilization of blood and blood components in one year (monthly). WB: whole blood, RCC: red cell concentrate, FFP: fresh frozen plasma, PRC: platelet-rich concentrate, CRYO: cryoprecipitate

Supply showed some seasonal variation, with lesser units being delivered at the beginning of the year and the peak observed towards the end of the year. In Medicine and Paediatric departments, maximum FFP and PRC are utilised for dengue patients particularly from August to November (Table 4, Figure 4).

**Table 4:** Utilization of blood and blood components in one year (monthly). WB: whole blood, RCC: red cell concentrate, FFP: fresh frozen plasma, PRC: platelet-rich concentrate, CRYO: cryoprecipitate

Months	WB	RCC	FFP	PRC	CRYO	Total
JANUARY	3	164	25	9	0	201
FEBRUARY	2	146	30	13	0	191
MARCH	1	208	51	20	0	280
APRIL	3	136	11	17	0	167
MAY	5	137	18	9	0	169
JUNE	6	168	16	14	0	204
JULY	4	264	37	26	0	331
AUGUST	8	210	148	88	0	454
SEPTEMBER	27	410	230	271	0	938
OCTOBER	7	515	447	441	0	1410
NOVEMBER	8	445	330	204	4	991
DECEMBER	10	311	91	63	0	475
TOTAL	84	3114	1434	1175	4	5811

**Figure 5:**
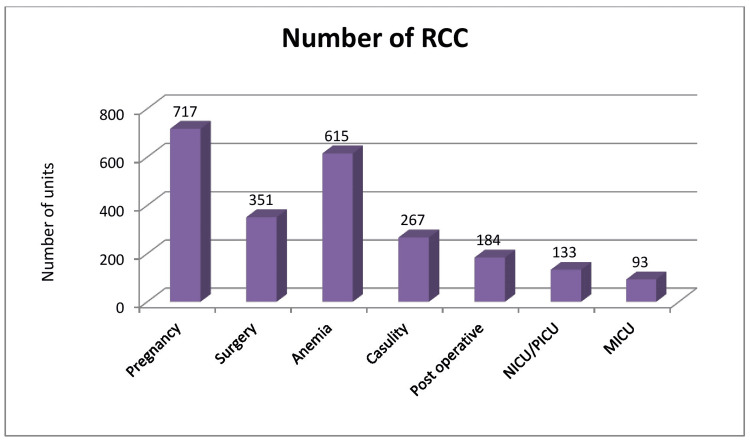
Comparison between various indications for most utilization of blood (RCC and WB n=2360) (*Exclude outside institute issue) WB: whole blood, RCC: red cell concentrate

**Figure 6:**
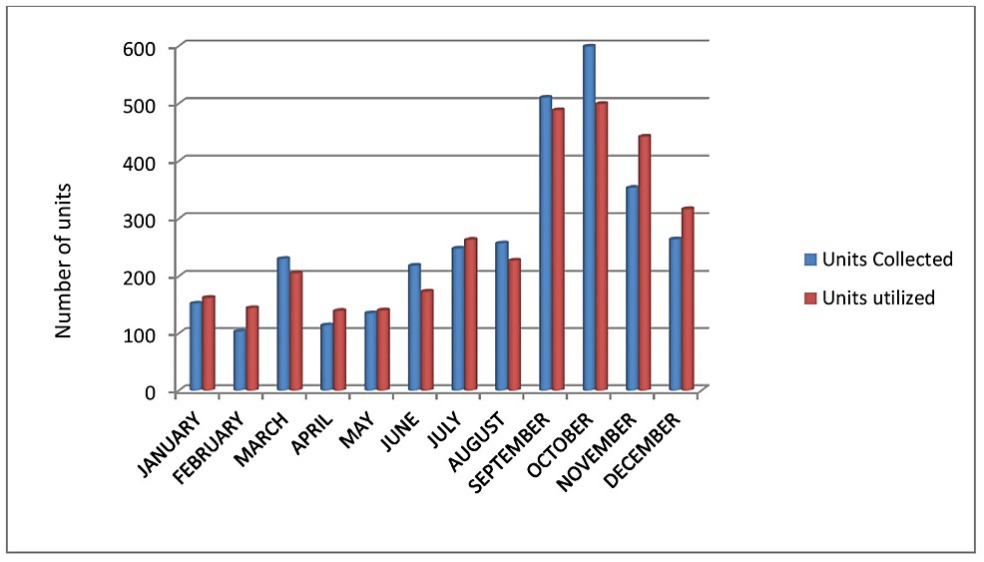
Blood units collected and utilized (WB and RCC) during the study period. WB: whole blood, RCC: red cell concentrate

The most common indication of red cell transfusion is pregnancy followed by anaemia (Table 5, Figure 5). As shown in Table 6 and Figure 6, the supply/demand of blood is more as compared to blood donations.

**Table 5:** Comparison between various indications for most utilization of blood (RCC and WB n=2360) (*Exclude outside institute issue) WB: whole blood, RCC: red cell concentrate

Cause	Number of Units	Percentages %
Pregnancy	717	30.38%
Surgery	351	14.87%
Anaemia	615	26.06%
Causality	267	11.31%
Post-operative	184	7.80%
NICU/PICU	133	5.64%
MICU	93	3.94%
Total	2360*	100%

**Table 6:** Blood units collected and utilized (WB and RCC) during the study period. WB: whole blood, RCC: red cell concentrate

Months	Units Collected	Units utilized
JANUARY	152	162
FEBRUARY	103	144
MARCH	230	205
APRIL	114	139
MAY	135	140
JUNE	218	173
JULY	248	263
AUGUST	257	227
SEPTEMBER	510	488
OCTOBER	599	499
NOVEMBER	353	442
DECEMBER	264	316
TOTAL	3183	3198

## Discussion

A blood transfusion service is an integral part of modern health care. Blood and blood components are considered drugs by the Food and Drug Control Authority (FDCA). Component therapy is designed to benefit multiple patients as the components prepared from a single unit of whole blood can serve various indications of transfusion needs. In recent years, the use of blood components has increased due to the burden of chronic diseases in the elderly population and blood-intensive surgical procedures [10]. This study describes the pattern of blood collection and utilization in a tertiary care hospital.

In the current study, we received 5811 requests and 5811 units components were issued after proper cross matching and screening. We noted 84 (1.54%) units of WB utilized among total 5811 blood units which is in contrary to the findings of Misal et al. [11] Dushyanth et al. [12] Girian et al. [13] and Joshi et al. [6] as they reported more whole blood utilization compared to other blood components. We maximize use of blood components instead of WB to achieve more benefits and reduce unwanted adverse reactions to patients. Anshoo et al. [14] and Venkatachalapathy and Subhashish [15] also found similar results as our study, which showed increased utilization of RCC among blood components. But Ambroise et al. [16] showed increased issue of FFP and platelets in relation to RCC. Ahmaed and Save [17] noted the highest utilization of RCC (74.9%) among paediatric patients while Venkatachalapathy and Subhashish noted increased utilization of blood units by the maternity department. In the present study, the majority of the blood units were issued to a gynecology department, which constituted 717 units among total 5811 units. This was followed by medicine and paediatrics departments which showed 460 and 320 of blood units respectively. For RCC and whole blood, most common indication was anaemia. Alcantara et al. [10] found the medicine department utilizing maximum number of blood components and a study conducted by Misal et al. showed that the most common indication for WB and RCC was anaemia which is similar to our study [11].

We optimize the use of blood components rather than whole blood to obtain more benefits and reduce adverse reactions in patients. RCC is the most frequently used product, followed by FFP and PRC. If we see the monthly distribution of blood components, FFP and PRC are most commonly used throughout the months of August through November. During this period, dengue fever was rampant throughout the district, making the PRC the most demanding product at the time. We see that the maximum component was utilized by medical departments and less in surgical departments. In the medical departments, RCC and WB are used for the treatment of anaemia whereas FFP and PRC are used for dengue fever and other coagulopathies.

Only the distribution of RCC and WB shows that the maximum number of RCC has been delivered to maternity, followed by medicine, paediatrics and casualty ward respectively. When it comes to RCC use, pregnancy is the most common indication, followed by anaemia and surgery. During pregnancy, the most frequent cause of blood transfusion is severe anaemia, followed by postpartum haemorrhage (PPH) and other complications.

The year 2021 shows that the utilization of RCC and WB (n=3198) is higher than the total blood donation (n=3183). This shows to what extent our region is in crisis.

It is very important to consider this when dealing with anaemia. It is important to note Hb and haematocrit values before blood transfusion and correlate the patient's clinical status [18]. Misuse of RCC may also be prevented in patients whose Hb or haematocrit may be improved through other means such as diet, haematin, etc. In maternity and medical departments, patients suffering from severe anaemia can be prevented by primary health centre (PHC) visits and early treatments in the early phase of anaemia.

Over the last two years, the use of PRC and FFP has increased due to their immediate availability, helping to manage acute haematological and coagulopathy conditions.

## Conclusions

The current study provides information on blood and blood component utilization pattern in our tertiary care hospital. Most blood requests (69.82%) came from Zydus Hospital, followed by 30.18% from other private hospitals. The major issued blood component was RCC (53.58%) followed by FFP (24.68%) and PRC (20.22%). The medical ward, followed by the obstetrics and paediatric wards of Zydus Hospital, made the most blood requests. The maternity ward majorly benefited from red cell concentrate. PRC and FFP were mainly used from September to November due to dengue fever. Anaemia was a major sign of blood requirements. Most RCCs can be used to correct anaemia, but surgical and traumatic use is low and caution is required. In addition, blood demand is very high compared to the total annual blood donation.

Every blood centre should audit the pattern of utilization of blood and blood components that can serve as an internal quality control for the effective functioning of the blood centre. Periodic reviews will also help in the formulation and implementation of standard guidelines for transfusion practices. Proper communication between clinicians and blood centre personnel will minimize inappropriate use of blood components. These efforts will meet some of the existing demand for blood components and prevent unwanted wastage of blood and blood components.
